# Supplementary material for: Novel Therapeutics for Type 2 Diabetes Mellitus—A Look at the Past Decade and a Glimpse into the Future
Source: Biomedicines. 2024 Jun 21;12(7):1386. doi: 10.3390/biomedicines12071386 (PMC11274090; doi:10.3390/biomedicines12071386)
Supplement: Supplementary file 1 [file biomedicines-12-01386-s001.zip › biomedicines-3014705-supplementary.pdf]

Supplementary data

Table S1: Selected studies evaluating the effect of SGLT2 inhibitors on physiological vascular measurements

Vascular measurement	Principle	Study design	Drugs tested	Comparator	Effect size	References
FMD	Well-recognized marker of endothelial function [27].	Meta-analysis	Dapagliflozin	Placebo or active comparator (not stated)	FMD increased by 1.66% (95% CI, 0.56 – 2.76)	[18]
		Meta-analysis	Dapagliflozin<br>Empagliflozin<br>Luseogliflozin<br>Tofoglflozin	Incretin<br>Metformin<br>Hydrochlorothiazide<br>Placebo	FMD in SGLT2 inhibitors groups significantly higher than controls with standardized mean difference (SMD): 0.18, (95% CI 0.02 – 0.34)	[28]
		Meta-analysis	Empagliflozin<br>Dapagliflozin	Sulphonylureas	SGLT2 inhibitors significantly increased FMD compared to sulphonylureas with a mean difference on 1.89 (95% CI 0.1 – 3.75)	[29]
EndoPAT	Assesses microvascular function through applying reactive hyperaemia on fingers [32]	RCT	Dapagliflozin	No dapagiflozin	LnRHI increased from 0.45 to 0.66, p<0.01 in the dapagliflozin group only	[32]
Pulse wave velocity (PWV)	Assesses arterial stiffness. A 1m/s increase in PWV is associated with 7% increased risk of cardiovascular event [33]	Meta-analysis (2023)	Dapagliflozin<br>Empagliflozin	Placebo	SGLT2 inhibitors reduced PWV by mean difference 0.76 (95% CI – 1.45 to -0.08)	[34]
		Observational	Tofogliflozin	-	Brachial-ankle PWV increased in the non-tofogliflozin group but not in the tofogliflozin group. Intergroup difference 100.2cm/s, p=0.018)	[35]

Table S2: Selected studies evaluating the effects of SGLT2 inhibitors on inflammatory, oxidative stress and thrombosis markers

Vascular marker	Function	Study design	Drugs tested	Comparator	Effect size	References
Inflammation						
CRP	Classic proinflammatory cytokine [41]	Meta-analysis	SGLT2 inhibitors	Conventional anti-diabetic drugs or placebo	CRP and ferritin levels were reduced in SGLT2 inhibitor groups (SMD: 0.25; 95% CI, -0.47, -0.03 and SMD: -1.21; 95% CI -1.91, -0.52) respectively	[41]
IL-6	Inflammatory cytokine that can accelerate atherosclerosis [19]	Meta-analysis	Dapagliflozin Empagliflozin Canagliflozin	Placebo or conventional anti-diabetic drugs	SMD of IL-6 was -1.04 (95% CI, -1.48 to -0.60 lower with SGLT2 inhibitors use. Dapagliflozin reduced IL-6 most by 1.3 (95% CI, -1.89 to -0.71)	[19]
IL-1β	Inflammatory cytokine stimulated by inflammasome and mediates atherosclerosis [38]	RCT	Empagliflozin	Placebo	Reduction in IL-1B by 4.58 pg/ml (95% CI -8.76, -0.41)	[21]
		RCT	Dapagliflozin	Placebo	IL-1β was lower at 356.4 ng/ml for dapagliflozin group vs 497.5 ng/ml for control group (p<0.001)	[40]
		RCT	Empagliflozin	Glimepiride	Reduction in macrophage secreted IL-1B reduced after SGLT2i from 3733 pg/ml to 2549 pg/ml vs 3777 pg/ml to 3121 pg/ml for glimepiride (p < 0.002)	[39]
TNF-alpha	Inflammatory cytokine that mediates pathways in atherosclerosis [39]	RCT	Dapagliflozin	Placebo	Reduction in TNF-α from 48.8 ng/L to 28.1 ng/L for dapagliflozin group vs 49.3 ng/L to 35.3 ng/L (p<0.001)	[40]
Oxidative stress						
Mitochondrial superoxide dismutase	Antioxidant enzyme that catalyses superoxide radicals [47].	Observational	Empagliflozin	Nil	Mitochondrial superoxide tended to reduce after 24 weeks of empagliflozin (p = 0.059)	[49]
		RCT	Empagliflozin	Placebo	Empagliflozin increased superoxide dismutase activity by 3.7 U/ml (95% CI 1.36, 6.05)	[21]

urine 8-hydroxy-2′-deoxyguanosin (8-OHdG)	Metabolite that reflects oxidative damage of DNA [27]	RCT	Dapagliflozin	Metformin	Reduction in 8-OHdG by 0.6 (p<0.001) vs increase by 1.1 in the metformin group	[27]
Thrombosis						
CD-62-P	Platelet activation marker stored in platelet granules and endothelial cells that is rapidly mobilised to the cell surface upon activation [55]	RCT	Empagliflozin	Placebo	Reduction in CD62-P Ag expression by 8.81 (95% CI -14.87, -2.75)	[21]
		Observational	Dapagliflozin	Nil	Dapagliflozin lowered CD62P-positive platelet counts (88.1 vs 77.6, p<0.05) without affecting haemostasis	[54]

Table S3: Selected studies evaluating the effect of SGLT2 inhibitors on cardiac biomarkers

Cardiac marker	Function	Study design	Drugs tested	Comparator	Effect size	References
NT-proBNP	Independent marker of ventricular wall stress, cardiac remodelling and dysfunction [57]	RCT	Dapagliflozin	Placebo	Dapagliflozin showed significant reduction in NT-proBNP of 18.2% (95% CI: - 27.1, - 8.2)	[23]
		RCT	Canagliflozin	Placebo	Median % change in NT-proBNP between canagliflozin and placebo were -11.9% (95% CI -18% to -5.6%) and -10% (95% CI -17.3 to -2.6%) at weeks 52 and 104	[24]
High-sensitivity troponin I (hs-TnI)	Measures of subclinical cardiac damage [61]	RCT (post-hoc analysis)	Empagliflozin	Placebo	Empagliflozin significantly reduced hs-TnI by 13.2, 95% CI -14.1 to – 12.3, p<0.001) after 26 weeks compared to placebo	[22]

Table S4: Selected studies of SGLT2 inhibitors and renal biomarkers

Renal marker	Function	Study design	Drugs tested	Comparator	Effect size	References
Urinary nephrin	Transmembrane glycoprotein located in the podocytes of the kidneys that serves as an early marker of glomerular damage in diabetic nephropathy [67]	RCT	Dapagliflozin empagliflozin canagliflozin	Comparison among the SGLT2 inhibitors	The urinary nephrin levels were significantly reduced in the microalbuminuria and macroalbumiuria groups compared to baseline in patients on SGLT2 inhibitors from 1.1 to 0.71 μg/ml (p=0.022) and 1.29 to 0.93 μg/ml (p=0.002)	[67]
Urinary liver-type fatty acid-binding protein (L-FABP)	Intracellular fatty acid carrier protein that reflects renal tubulointerstitial damage [73]	RCT	Cannagliflozin	Usual care	Reduction in urinary L-FABP by 65% (95% CI, -70 to – 38) in the canagliflozin group but not in the control group.	[74]
Kidney injury molecule 1 (KIM-1)	Membrane protein expressed in the kidney that reflects renal tubular injury [76]	RCT (post-hoc analysis)	Ertuglliflozin	Placebo	Ertugliflozin use was associated with approximately 20% reduction in KIM-1 (p=0.007)	[77]
		RCT (post-hoc analysis)	Dapagliflozin	Placebo	Dapagliflozin reduced urinary KIM-1 excretion by 22.6% (95% CI, 0.3 – 39.8)	[78]

Table S5: Selected studies of GLP1-RAs and the effects on physiological vascular measurements

Vascular measurement	Function	Study design	Drugs tested	Comparator	Effect size	References
FMD	Well-recognized marker of endothelial function [27]	Meta-analysis	GLP-1 RAs	Lifestyle intervention sulphnoylureas placebo	GLP-1 RAs improved FMD by mean difference 3.7 (95% CI, 1.39 – 5.97) compared to lifestyle intervention. GLP-1 RAs improved FMD by mean difference 3.33 (95% CI, 1.36 – 5.34) and 3.30 (95% CI, 1.21 – 5.43) compared to sulphonylureas and placebo, respectively	[29]
PWV	Assesses arterial stiffness [33]	Meta-analysis	GLP-1 RAs	Placebo	In patients with abnormal glucose metabolism, GLP1-RAs reduced PWV by mean difference of 1.06 (95% CI, -2.05 to -0.10)	[34]

Table S6: Effects of GLP-1 RAs on vascular and cardiac biomarkers

Vascular marker	Function	Study design	Drugs tested	Comparator	Effect size	References
Inflammation						
CRP,<br>TNF-α	Classic proinflammatory cytokines that mediate atherosclerosis [39]	Meta-analysis (n=6749)	GLP-1 RAs	Standard therapies or placebo	GLP-1 RAs reduced CRP by mean difference of -0.54 mg/L (95% CI, -0.75 to -0.34] and TNF-α by mean difference 0.39 (95% CI, -0.62 to -0.15].	[94]
MCP-1	Promotes recruitment of monocytes and macrophages to the subendothelial layer, driving atherosclerosis [100]	RCT	Liraglutide	Sitagliptin or placebo	Liraglutide reduced MCP-1 by 10.7 (95% CI, -17.7 to -3.7) pg/ml at 14 weeks vs no change with sitagliptin	[95]
VCAM-1	Endothelial cell surface adhesion molecule that promotes monocyte migration across vessel wall to drive atherosclerotic lesion formation [101]	RCT	Liraglutide	Metformin	Significant reduction in VCAM-1 from 503 to 382 ng/ml (p=0.046) in liraglutide group versus an increase in VCAM-1 in metformin group	[102]
Oxidative stress						
Malondialdehyde (MDA)	Derived from lipid peroxidation and serves as a marker of oxidative stress [104]	Meta-analysis (n=6749)	GLP-1 RAs	Standard therapies or placebo	GLP-1 RAs reduced MDA by mean difference of -0.84 (95% CI, -1.61 to -0.06]	[94]
Thrombosis						
PAI-1	Key regulator of fibrinolysis that is tightly linked to the atherosclerotic process [97]	RCT	Liraglutide	Sitagliptin or placebo	Liraglutide reduced PAI-I by 3.7 (95% CI, -5.5 to -2.0) U/ml at 14 weeks vs no change with sitagliptin	[95]
Cardiac marker						
NT-proBNP	Independent marker of ventricular wall stress, cardiac remodelling and dysfunction [57]	RCT	Liraglutide	Metformin	Significant reduction in NT-pro-BNP from 432 to 282 pg/ml (p=0.03) vs nonsignificant change in metformin	[112]

Table S7a: Completed trials for tirzepatide in T2DM patients

Trial	Sample size / additional criteria	Comparators	Findings
SURPASS-1 [151]	N = 705<br>Inadequately controlled by diet and lifestyle modifications	Tirzepatide 5mg vs 10mg vs 15mg vs placebo for 40 weeks	Dose dependent reduction in HbA1c by 2.07% with tirzepatide 15mg vs +0.04% with placebo
SURPASS-2 [152]	N = 1879	Tirzepatide at 5mg, 10mg or 15mg vs semaglutide 1mg for 40 weeks	Tirzepatide 15mg reduced Hb1c by 0.45% and weight by 5.5kg more than semaglutide
SURPASS-3 [153]	N = 1444<br>BMI ≥ 25<br>Only on metformin +/- SGLT2 inhibitor	Tirzepatide 5,10 or 15mg vs degludec for 52 weeks	Tirzepatide 15mg reduced HbA1c by 2.37% vs 1.34% for degludec. Weight reduced by 12.9kg for tirzepatide 15mg vs increased 2.3kg for degludec.
SURPASS-4 [154]	N = 2002	Tirzepatide 5,10, 15mg vs glargine for 52 weeks	Tirzepatide 15mg reduced HbA1c by 2.58% vs 1.44% with glargine. Estimated treatment difference was 0.99%.
SURPASS-5 [155]	N = 475<br>On glargine	Tirzepatide 10mg or 15mg vs placebo as add-on to glargine for 40 weeks	Addition of tirzepatide 10mg and 15mg led to 2.4% and 2.34% reduction in HbA1c
SURPASS-6 [233]	N = 1428<br>On basal insulin	Tirzepatide 5mg, 10mg, 15mg vs lispro TDS for 52 weeks	HbA1c for pooled tirzepatide cohort was reduced by 2.1% vs 1.1% for lispro
SURPASS AP COMBO [234]	N = 917<br>On metformin +/- sulphonylurea	Tirzepatide 5mg, 10mg, 15mg vs glargine for 40 weeks	Reduction in HbA1c by 2.49% with tirzepatide 15mg vs 0.95% with glargine<br>Weight loss of 7.2kg for tirzepatide 15mg va weight gain 1.5kg for glargine
SURMOUNT 2 [235]	N = 938<br>BMI ≥ 27	Tirzepatide 10mg, 15mg vs placebo for 72 weeks	Body weight reduced by 14.7% with tirzepatide 15mg vs 3.2% reduction with placebo

Table S7b: Tirzepatide trials for obesity

Trial	Sample size	Comparators	Findings
SURMOUNT-1 [156]	N = 2359	Tirzepatide 5,10,15mg vs placebo for 72 weeks	Tirzepatide 15mg reduced weight by 20.9% compared to 3.1% for placebo

	BMI ≥ 30 or ≥ 27 with weight-related complications		
SURMOUNT-4 [236]	N = 670<br>BMI ≥ 30 or ≥ 27 with weight-related complications	All participants received tirzepatide for 36 weeks and then randomized to continue tirzepatide or switch to placebo for 52 weeks	At least 80% who received tirzepatide during lead-in period maintained weight loss compared with 16.6% receiving placebo with overall weight reduction of 25.3% for tirzepatide and 9.9% for placebo.

Table S7c: Ongoing tirzepatide trials

Trial	Population / sample size	Comparators	Findings
SURPASS SWITCH (NCT05564039)	N = 250<br>T2DM on stable dose of dulaglutide	Switching dulaglutide to tirzeparide vs increasing dulaglutide dose for 40 weeks	Change in HbA1c
SURPASS SWITCH 2 (NCT05706506)	N = 152<br>T2DM already on a GLP-1 RA	Switching GLP-1 RA to tirzepatide for 12 weeks for 104 weeks	Change in HbA1c
SURPASS early (NCT05433584)	N = 780<br>T2DM diagnosed within last 4 years and only on metformin	Tirzepatide vs intensified conventional care	Change in HbA1c
NCT06037252	N = 350<br>T2DM and obesity on metformin	Addition of tirzepatide vs placebo for up to 80 weeks	Change in HbA1c and body
NCT06221969	N = 1000<br>T2DM and BMI ≥ 30	Cagrilintide and semaglutide combination vs tirzepatide for 68 weeks	Change in HbA1c and weight
NCT06131347	N = 800<br>BMI ≥ 30 and HbA1c ≥ 6.5%	Cagrilintide and semaglutide combination vs tirzepatide for 72 weeks	Change in body weight
NCT04255433	N - 13299<br>T2DM with ASCVD	Tirzepatide vs dulaglutide	Time to first occurrence of cardiovascular mortality or MACE
TREASURE CKD (NCT05536804)	N = 140	Tirzepatide vs placebo	Change in kidney oxygenation (using MRI)

	BMI ≥ 27 with eGFR < 50 ml/min/1.73m2 or eGFR 25-75 with UACr > 30mg/g and already on ACE or ARB<br>With or without T2DM		
SUMMIT (NCT04847557)	N = 731<br>Stable heart failure with NYHA class II-IV and LVEF > 50%<br>Elevated NT pro BNP<br>BMI > 30	Tirzepatide vs placebo for 52 weeks	Change in Kansas City Cardiomyopathy Questionnaire Clinical Summary Score and occurrence of composite endpoint of CV mortality or heart failure events

Table S8: Selected ongoing early phase or recently completed trials involving novel antidiabetic agents

Class	Drug	Selected ongoing early phase or recently completed trials					
		Sample size	Sample population	Comparator	Duration	Outcome	Phase
Amylin analogue	Cagrilintide (NCT06065540)	2700	T2DM with HbA1c 7-10.5% and BMI > 25	Placebo or semaglutide	66 weeks	Change in HbA1c and body weight	3
Glucokinase activator	Dorzagliatin [188]	463	T2DM drug-naive	Placebo	24 weeks	HbA1c was 1.07% lower (p<0.001) in dorzagliatin group	3
	TTP399 (AGATA trial) Results published [197]	190	T2DM with HbA1c between 7-9.5% on metformin	Placebo	6 months	Reduction in HbA1c by 0.9% compared to placebo (p<0.01)	2
	PB201 (NCT05102149) [198] (Du)	672	Treatment-naïve T2DM	Vildagliptin or placebo	24 weeks	Change in HbA1c	3
	PB201 (NCT05326490)	546	T2DM suboptimally controlled on metformin	Placebo	24 weeks	Change in HbA1c	3
Oral GLP1-RA	CT-996 (NCT05814107)	118	T2DM and BMI 25-40	Placebo	Not stated	Safety and tolerability	1
	XW-014 (NCT05579314)	104	T2DM 6.5 – 8.5%<br>BMI 30-40	Placebo	Not stated	Safety and tolerability	2
	GSBR-1290 (NCT05762471)	142	T2DM HbA1c 7 – 10.5% on metformin<br>BMI 27 - 40	Placebo	Not stated	Safety and tolerability	1/2
	Orforglipron (LY3502970) NCT06192108	888	T2DM with HbA1c 7 – 10.5% on metformin	Dapagliflozin	40 weeks	Change in HbA1c	3
Subcutaneous GLP1-RA	GZR-18 (NCT06256549)	272	T2DM with HbA1c 7 – 11% on stable doses of oral hypoglycaemic agents	Placebo	24 weeks	Reduction in HbA1c	2
	Ecnoglutide [199]	206	BMI 30 – 40	Liraglutide 3mg daily	26 weeks	Reduction in body weight of 14.7% (receiving highest	2

						dose of ecnoglutide) vs 8.8% for liraglutide	
	Supaglutide [200]	340	T2DM inadequately controlled on metformin	Placebo	24 weeks	Greater reduction in HbA1c by 1.81% (95% CI, -2.02 to -1.6) vs placebo -0.757 (95% CI, -1 to -0.52)	3
GLP-GIP	HRS9531 (NCT05516966)	Not stated	T2DM 7-10.5% - treatment naïve or only on metformin	Dulaglutide	Not stated	Safety and tolerability	1
	CT-388 (published as abstract) (Chakravarthy) [201]	64	Overweight or obese	No	4 weeks	Pharmacokinetic profile, safety and tolerability	1
	HS-20094 (NCT06118008)	96	T2DM 7.5 – 10% on stable dose of metformin	Placebo or semaglutide	Not stated	Safety and tolerability	2
	AMG 133 (NCT05669599)	592	T2DM with HbA1c 7 – 10% BMI ≥ 30 or ≥ 27 with at 1 obesity-related comorbidity	Placebo	Not stated	Reduction in HbA1c	2
GLP-glucagon receptor agonist	DD01 (NCT04812262)	255	T2DM with HbA1c < 10% on diet / lifestyle modifications or metformin<br>BMI between 25 - 40<br>NAFLD determined by MRI	Placebo	Not stated	Safety and tolerability	1
	IBI362 (LY3305677) – results published [202]	12	BMI ≥ 24 with at least one obesity-related comorbidity or BMI ≥ 28	Placebo	12 weeks	Gastrointestinal intolerance and reduced appetite are the most common. 3 subjects had mild and asymptomatic cardiac disorders revealed by ECG	1b
	IBI362 (mazdutide) (NCT06184568)	342	T2DM ≤ 5 years with HbA1c 7.5 – 9.5% controlled on diet	Semaglutide	40 weeks	Proportion of subjects achieving HbA1c <	3

			and lifestyle modifications or metformin BMI ≥ 28			7% and weight loss ≥ 10%	
	BI 456906 (SYNCHRONIZZE-1) (NCT006066515)	600	BMI ≥ 30 or BMI ≥ 27 with at least one weight-related problem	Placebo	76 weeks	Percentage change in body weight up till 76 weeks	3
Triple agonist (retratrutide)	TRIUMPH 2 (NCT05929079)	1000	T2DM on stable treatment and OSA	Placebo	80 weeks	Percent change in body weight and apnea-hypopnea index	3
	TRANSCEND-T2D-2 (NCT06260722)	1250	T2DM - HbA1c 7-10.5% on stable metformin dose with or without SGLT2 inhibitor BMI ≥ 25	Semaglutide	26 months	Change in HbA1c	3
	TRANSCEND-T2D-3 (NCT06297603)	320	T2DM – HbA1c ≥ 7% with moderate or severe renal impairment on stable dose of basal insulin with or without metformin and/or SGLT2 inhibitor	Placebo	14 months	Change in HbA1c	3
Glimin	Imeglimin (NCT05366868)	567	T2DM only on diet and lifestyle modifications with HbA1c 7-9%	Metformin or vildagliptin	156 weeks	Time to achieve 2 consecutive HbA1c ≥ 7%	4
GPR40	CPL207280 (published) [196]	56	Healthy volunteers	None	Not stated	Safe and well tolerated	1
	IDG-16177 (NCT04982705)	94	Healthy volunteers	Placebo or sitagliptin	Not stated	Safety and tolerability	1
	HD-6277 (NCT05666128)	113	T2DM with HbA1c 7-10% on diet and lifestyle modifications only	Placebo	Not stated	Change in HbA1c	2
Insulin sensitiser (PPAR agonist)	MSDC-0602K (NCT03970031)	1800	PreDM or T2DM with NAFLD	Placebo	26 weeks	Change in HbA1c, liver enzymes	3
	Chiglitazar (NCT04807348) Results published [203]	519	T2DM with HbA1c 7.5 – 11% on metformin	Placebo	24 weeks	Reduction in HA1c by 1.05% (95% CI -1.29 to -0.81) for highest	3

						dose of chiglitazar compared to placebo	
	Chiglitazar (NCT05760677)	142	T2DM and polycystic ovarian syndrome	Lifestyle intervention or metformin or orlistat	3 months	Change in plasma glucose	1
